# Homelessness Is Socially Created: Cluster Analysis of Social Determinants of Homelessness (SODH) in North West England in 2020

**DOI:** 10.3390/ijerph18063066

**Published:** 2021-03-16

**Authors:** Mzwandile Mabhala, Winifred Adaobi Esealuka, Amanda Nkolika Nwufo, Chinwe Enyinna, Chelsea Nonkosi Mabhala, Treasure Udechukwu, John Reid, Asmait Yohannes

**Affiliations:** 1 Faculty of Health and Social Care, Department of Public Health and Wellbeing, University of CHESTER, Riverside Campus, Chester CH1 1SL, UK; winifred.esealuka@yahoo.com (W.A.E.); amandanwufodr@gmail.com (A.N.N.); chinweenyinna@gmail.com (C.E.); john.reid@chester.ac.uk (J.R.); 2 Faculty of Sciences, School of Pharmacy, University of East Anglia, Norwich, Norfolk NR4 7TJ, UK; cmabhala@gmail.com; 3 École des Hautes Études en Santé Publique, 93 210 Paris, France; treasure.udechukwu@eleve.ehesp.fr; 4 Asmait Skincare and Design, Stamford, CT 06902, USA; asmaitskincare@gmail.com

**Keywords:** homelessness, poverty, inequalities, two-step cluster analysis

## Abstract

Poverty creates social conditions that increase the likelihood of homelessness. These include exposure to traumatic life experiences; social disadvantages such as poor educational experiences; being raised in a broken family, care homes or foster care; physical, emotional, and sexual abuse; and neglect at an early age. These conditions reduce people’s ability to negotiate through life challenges. This cross-sectional study documents the clustering and frequency of adverse social conditions among 152 homeless people from four cities in North West England between January and August 2020. Two-step cluster analysis showed that having parents with a criminal record, care history, and child neglect/abuse history was predictive of homelessness. The cluster of indicator variables among homeless people included sexual abuse (χ2 (N = 152) = 220.684, *p* < 0.001, Cramer’s V = 0.7), inappropriate sexual behaviour (χ2 (N = 152) = 207.737, *p* < 0.001, Cramer’s V = 0.7), emotional neglect (χ2 (N = 152) = 181.671, *p* < 0.001, Cramer’s V = 0.7), physical abuse by step-parent (χ2 (N = 152) = 195.882, *p* < 0.001, Cramer’s V = 0.8), and physical neglect (χ2 (N = 152) = 205.632, *p* < 0.001, Cramer’s V = 0.8). Poverty and homelessness are intertwined because of the high prevalence of poverty among the homeless. Poverty sets up a chain of interactions between social conditions that increase the likelihood of unfavourable outcomes: homelessness is at the end of the interaction chain. Interventions supporting families to rise out of poverty may also reduce entry into homelessness.

## 1. Background 

Globally, homeless people are materially poor. Homelessness is higher among those who have had exposure to traumatic life experiences, poor schooling experiences, or disruptive families; were raised in children’s care homes or foster care; were physically, emotionally, or sexually abused; or suffered neglect at an early age [1,2,3,4,5,6,7,8]. In this study, we adopted Early Intervention Foundation’s [9] definition of adverse childhood experiences (ACEs) which is “traumatic events or circumstances occurring before the age of 18 ([9], p. 6)”. The original 10 ACEs were reported by Dube et al. [10]. They are: physical abuse, sexual abuse, psychological abuse, physical neglect, psychological neglect, witnessing domestic abuse, having a close family member who misused drugs or alcohol, having a close family member with mental health problems, having a close family member who served time in prison, and parental separation or divorce on account of relationship breakdown. 

The current understanding is that the causes of homelessness are complex. Several researchers broadly categorise them into individual factors and structural factors [11,12,13,14,15]. Individual factors are associated with individual circumstances or behaviours that could increase a person’s vulnerability to homelessness. Examples that emerged from the literature include adverse childhood experiences (ACEs); mental health poor physical health; substance misuse problems; experience of domestic violence, abuse, neglect, harassment or hate crime, bereavement, and relationship breakdown; experience of care or prison; refugees; and association with criminal justice systems [13,14,16]. Structural factors are associated with social policy, society, and social institutions that create and sustain social conditions that cause homelessness [13,14,17]. The proponents of structural factors argue that homelessness is a socially caused phenomenon. Examples include unevenly distributed, inadequate or absent low-cost housing, educational and employment opportunities, income support, and social benefits [13].

The consensus exists that poverty is a common factor associated with both individual and structural causes of homelessness [16,17,18,19,20]. However, there are different views on how they are associated. Some appear to suggest that poverty’s harm is due to the lack of material resources; their premise is that providing material things that people lack, such as food and permanent accommodation, would prevent homelessness [21,22,23]. Others appear to suggest that poverty’s harm is due to its effect on social disadvantages such as inability to access educational and employment and income opportunities; exposure to physical, emotional, or sexual abuse; and adopting maladaptive behaviours [19,24,25,26]. These differences shape the debate about whether the policy on homelessness should focus on tackling poverty as a fundamental cause of homelessness, or on immediate causes and harmful effects of homelessness [13,15,19,20].

We found no studies that specifically examined the causative relationship between ACE and homelessness. However, the number of studies in which these conditions exist concurrently makes it plausible to conclude that they are connected. For example, several studies indicated that people who have had exposure to ACEs are less likely to adapt successfully than people without such exposure [3,4,27,28,29,30]. Furthermore, studies revealed that people exposed to ACEs are more susceptible to adopting maladaptive coping behaviours such as theft, trading sex for money, and selling or using drugs and alcohol [31].

Several studies have examined the effects of specific childhood traumatic experiences on social indicators of homelessness such as low educational achievement, unemployment, and maladaptive behaviours [7,32,33,34,35]. For example, Spinelli, Ponath et al. [35] examined the prevalence of and the factors associated with ACEs in a population-based sample (*n* = 350) of homeless individuals aged 50 and older in Oakland, CA, USA. The study showed that all homeless people in the survey had severe adverse childhood experiences: a third (31.2%) of the older homeless people had experienced suspension or expulsion from school and a third (33.3%) experienced physical abuse as a child. In comparison, 13.2% experienced sexual abuse as a child, half (51.2%) experienced physical violence as an adult, and 13.2% experienced sexual abuse as an adult [7].

Barker, Kerr et al. [32] examined the relationship between five categories of childhood maltreatment (physical, emotional, and sexual abuse and physical and emotional neglect) and completion of high school education amongst the homeless. After adjustment for confounding variables, the analysis indicated that four forms of maltreatment remained significantly and independently associated with not completing a high school education: physical abuse; emotional abuse; physical neglect; and emotional neglect. Similarly, Patterson et al. [36] reported that having a history of foster care placement independently predicted incomplete high school education, duration of homelessness, discontinuous work history, less severe types of mental illness, multiple psychiatric disorders, early initiation of drug and alcohol use, and daily drug use.

Some combinations of ACEs are more common amongst the homeless than others [30,36,37,38]. Mental health and behavioural disorders, poor school performance, a history of foster care and disrupted family structure were most associated with adult criminal activities, adult substance use, unemployment and subsequent homelessness [30,36,37,38].

This cross-sectional study aimed to document the clustering and frequency of adverse social conditions among homeless people in North West England in 2021. To the best of our knowledge based on several years of studying the determinants of homelessness, the current study is the first that statistically models the clusters of social conditions predictive of homelessness.

## 2. Methods

### 2.1. Study Design

A cross-sectional design was considered the most appropriate to address this project’s aim and objectives, as it is particularly useful for establishing prevalence and identifying underlying risk factors [39,40].

We used a simple random sampling approach to ensure that each homeless population member had an equal chance of being selected. We presumed that each participant represents a typical homeless person in Cheshire, Liverpool, or Manchester, and thus can be generalised directly to that population [40]. Such approach minimises the risk of selection bias [40].

### 2.2. Study Population

The studied population were homeless people. For this study, we defined homelessness as not having a permanent home. We recognised that the definition of homelessness varies according to the context within which it occurs. Our definition is consistent with the UK legal definition of homelessness; which is” that a household has no home in the UK or anywhere else in the world available and reasonable to occupy [41].” Homelessness does not just refer to people who are sleeping rough. Since we had no way to validate our participants’ status, we regarded all individuals who declared themselves as homeless and were accessing homeless people’s facilities for a place to stay, safety, food, and healthcare as homeless. Shelter—one of the largest UK homeless charities contends that you may be homeless if you’re sleeping rough, don’t have rights to stay where you are, or you live in unsuitable housing [42].

### 2.3. Sample Size and Data Collection

One hundred and fifty-two homeless people completed the survey. This study sample was higher than the calculated sample size effect of a 137, based on the December 2019 North West England estimated homeless population of 9038 [43]. We used OpenEpi 3.01 to calculate the sample size.

We collected data from homeless people in Chester (57), Crewe (3), Liverpool (57), and Manchester (35) between January 2020 and August 2020. In Chester, the data was collected in two facilities, in Crewe one facility, in Liverpool in two facilities and three facilities in Manchester. The principal investigator has researched homeless for several years; we have learned from previous studies that some homeless people have a limited level of literacy. We, therefore, decided to administer the questionnaires face to face.

The Faculty of Health and Social Care Research Ethics Subcommittee at the University of Chester granted ethical approval of this study.

### 2.4. Data Variables Collected

Table 1 summarises the study’s objectives and corresponding variables measured and developed based on previous studies. We hypothesised that the variables’ observed frequencies would be different amongst the homeless from expected in all five objectives and associated social exposure variables. We also hypothesised that some social variables clusters would have a higher predictor of importance than expected.

### 2.5. Analysis and Statistics

We used IBM SPSS, statistical package version 21(New York, NY, USA), with a significance level set at *p* < 0.05 and confidence interval at 95%, to carry out chi-square (χ2) goodness of fit tests, two-step cluster analysis, and Bonferroni and Cramer’s V post hoc tests.

We used the χ2 test to determine how the observed frequencies of homelessness indicators are significantly different from the expected values [44]. To protect ourselves from committing type 1 error, i.e., declaring that the difference between the observed and expected frequencies is significant when it is not, we conducted a series of post hoc χ2 analyses using the Bonferroni correction.

Furthermore, to establish the strength of association amongst the variable indicators, we conducted Cramer’s V post hoc analysis. Cramer’s V was chosen because it is a preferred measure of the effect size of the χ2 [45]. We used Cohen’s effect size guidelines for the interpretation of the Cramer’s V findings [45,46].

Finally, we conducted a two-step cluster analysis to classify variable indicators according to their predictor of importance [47,48]. We decided to use the two-step cluster analysis procedure because it is compatible with categorical and continuous variables. Consistent with χ2 and Cramer’s V, two-step cluster analysis assumes that the cluster model variables are independent [47].

## 3. Results

### 3.1. The Description of the Health and Demographic Characteristics of Homeless People

Table 2 describes the characteristics of study participants according to the different locations where the data was collected. One hundred and fifty-two homeless people with a mean age of 39 years participated in this study. There were more males (63%) than females (37%); mostly unmarried (91%); and living in the street (31%), temporary accommodation (TA) (21%), or hostels (19%).

Among them, 86% of the participants smoked tobacco products daily, and 80% used drugs daily. The most commonly used drugs were marijuana (58%), cocaine (43%), heroin (34%), and spice (19%).

Furthermore, 61.2% described their health as poor, and 69% reported that they had seen a medical doctor in the last 6 weeks.

### 3.2. The Social Characteristics of the Parents of Homeless People

We hypothesised that the observed social status indicators amongst homeless people’s parents would differ from expectations. The calculated χ2 of the following variables were greater than critical values (*p* ≤ 0.001): highest educational attainment, marital status, employment status, criminal record, care history, and history of child neglect/abuse.

However, the χ2 tests did not provide any indication of the order of importance. Therefore, we conducted a two-step cluster analysis to classify these indicators according to their predictor of importance. Parsimony principles guided our cluster analysis, which meant that we carried out multiple iterations and chose the simplest model that fitted the evidence [49]. The best fit solution is indicated in Table 3; it shows the model ratio of size, average silhouette, predictor importance values, cluster χ2 tests values, and variable-specific Cramer’s V values.

The best fit cluster solution identified three indicators of the social status of homeless people’s parents—criminal record, care history, and child neglect/abuse history. The two-step analysis classified these into three clusters with the indication of quality as “good,” average silhouette measure of 0.8 [50] and the ratio of size equalling 5.10. Cluster 1 was the largest cluster with 67.1% (observed frequency *[fo]* = 106), followed by cluster 3 with 19.7% (*fo* = 30); the smallest was cluster 2 with 13.2% (*fo* = 20).

In terms of the order of predictor importance, the two-step cluster analysis detected that having parents with a criminal record was the highest (1.00), followed by parents with care history (0.89) and parents with child neglect/abuse history (0.72).

We performed χ2 goodness of fit tests to examine the significance of the differences amongst the predictor variables within the cluster. We also performed a post hoc test using the Bonferroni (correction) chi-square z-value analysis. The Bonferroni post hoc analysis revealed that the differences amongst the three PImp values remained significant: criminal record (χ2 (3, N = 152) = 231.105, *p* < 0.001, Cramer’s V = 0.9); care history (χ2 (2, N = 152) = 169.434, *p* < 0.001, Cramer’s V = 2); and history of child abuse or neglect (χ2 (2, N = 152) = 187.316, *p* < 0.001, Cramer’s V = 1.8).

We also examined the strength of the significance amongst the predictor variables with post hoc Cramer’s V, using the formula $V=\sqrt{\frac{\chi 2}{n\left(k-1\right)}}$. Based on Cohen’s effect size guidelines, there was significantly strong association amongst the variables, with Cramer’s V = 0.9, 2, and 1.8, respectively [45,46,47,51,52].

### 3.3. The Occurrence and Frequency of Adverse Clusters of Social Conditions among Homeless People

We wanted to determine how the homeless people’s childhood social conditions compared with the null hypothesis’s expectation. The χ2 goodness of fit test found statistically significant differences between the expected frequencies (*fe)* and the *fo* of childhood living arrangements (χ2 (11, N = 152) = 398.263158, *p* < 0.001); educational arrangement (χ2 (6, N = 152) = 432.868, *p* < 0.001); and highest qualification (χ2 (6, N = 152) = 319.669, *p* < 0.001).

Again, we performed Bonferroni post hoc analysis to examine the differences amongst the variable indicators. Table 4 shows that the observed adverse social conditions that were significantly higher than expected were being raised by single mother (χ2 (*fo* = 19) = 39.69, *p* < 0.001); living in care (χ2 (*fo* = 29) = 265.69, *p* < 0.001); and leaving school before 16 (χ2 (*fo* = 80) = 3410.56, *p* < 0.001) and at 16 (χ2 (*fo* = 61) = 1552.36, *p* < 0.001).

### 3.4. The Occurrence and Frequency of Adverse Childhood Experiences Amongst Homeless People

We performed χ2 goodness of fit tests to determine how the *fo* of ACEs amongst homeless people compared to that expected if the null hypothesis is true. These indicated that the frequency of ACEs amongst homeless people was significantly different. However, before rejecting the null hypothesis, we performed Bonferroni corrections to examine how the observed indicators of ACE differed from expectations. The analysis revealed that verbal abuse (χ2 (*fo* = 83) = 1043.29, *p* < 0.001), experience of threatening behaviour (χ2 (*fo* = 69) = 112,151.31, *p* < 0.001), and physical violence (χ2 (*fo* = 65) = 204.490, *p* < 0.001), were higher amongst homeless people than expected.

We performed a two-step cluster analysis to classify ACEs amongst the homeless according to their PImp. Following multiple iterations, the best cluster solution model detected was the one with three clusters. The ratio of size between the smallest and the largest was 3.8; cluster 2 was the smallest (16.4%) followed by cluster 1 (21.1%) and cluster 3 was the largest (62.5%).

In terms of predictor importance, sexual abuse (1.00), inappropriate sexual behaviour (0.99), emotional neglect (0.70), physical abuse by step-parent (0.65), and physical neglect (0.64) were the most important predictors. Cramer’s V post hoc test indicated that the following indicator variables are statistically significant: sexual abuse (χ2 (N = 152) = 220.684, *p* < 0.001, Cramer’s V = 0.7); inappropriate sexual behaviour (χ2 (N = 152) = 207.737, *p* < 0.001, Cramer’s V = 0.7); emotional neglect (χ2 (N = 152) = 181.671, *p* < 0.001, Cramer’s V = 0.7); physical abuse by step-parent (χ2 (N = 152) = 195.882, *p* < 0.001, Cramer’s V = 0.8); and physical neglect (χ2 (N = 152) = 205.632, *p* < 0.001, Cramer’s V = 0.8) (see Table 5).

### 3.5. The Factors Leading to Homelessness

Table 6 depicts a two-step analysis model that classifies the factors that led people to become homeless. The two-step cluster analysis and χ2 and post hoc tests revealed that the most significant factors were drug alcohol dependence, eviction due to criminal activities, loss of job, and being imprisoned.

## 4. Results Conceptual Summary

**Figure 1:**
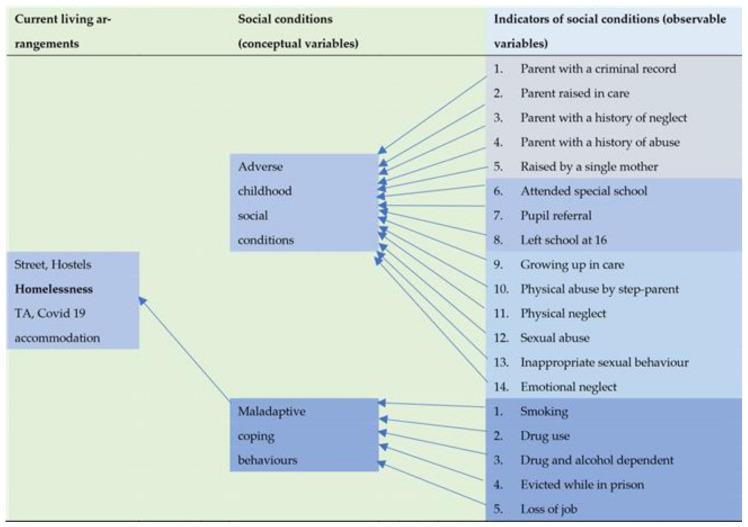
The current living arrangements, past social conditions, and indicators of social conditions (observable variables) relating to homeless people in the North West of England in 2020.

All participants in this study had no permanent home. Figure 1 indicates that participants lived in the street, hostels, temporary accommodation, and other accommodation provided during the COVID-19 pandemic. Figure 1 also illustrates the life-course approach to analysing the homeless people’s variables relating to their current and past social circumstances, including the circumstances they were born into, childhood experiences and adulthood circumstances.

Our analysis detected 19 observable indicators of social conditions amongst the homeless that were significantly different from expectations. Fourteen of them related to adverse childhood social conditions, and five related to adulthood maladaptive coping behaviour.

We constructed the 14 observable indicators of adverse childhood social conditions into parental characteristics (instability and disruption in the family), disruption in education, and maltreatment. The 14 indicators in this study were comparable with 10 indicators of ACEs identified by the CDC Kaiser-ACEs study [53], one of the largest US-based studies on ACEs. They were also similar to those reported by Lacey et al. [54], one of the largest UK longitudinal studies of parents and children ACEs.

There was a consensus amongst all studies that most adverse childhood experiences have their roots in poverty [54,55,56]. Being poor is associated with so many childhood adversities that it may be considered an ACE in itself. Hughes and Tucker [56] argue that poverty acts as a reinforcing mechanism, disproportionately burdening low-income families with stressors that give rise to adverse conditions. Several studies found that poverty was linked to most of the indicators of adverse childhood conditions identified in our study, including low educational attainment, parental criminal record, and increased likelihood of being subject to abuse and neglect [53,54,55,56]. Furthermore, the study by Lacey et al. [54] showed a strong association between poverty, sexual abuse (OR = 2.38, 95% CI = [1.62, 3.52]), and parental separation (OR = 2.63, 95% CI = [2.20, 3.14]).

This study adopted Townsend’s [57] definition of poverty which is:

“Individuals, families and groups in the population can be said to be in poverty when they lack the resources to obtain the types of diet, participate in the activities and have the living conditions and amenities which are customary or are at least widely encouraged or approved, in the societies to which they belong. Their resources are so seriously below those commanded by the average individual or family that they are, in effect, excluded from ordinary living patterns, customs and activities.”

(Townsend, 1979, p. 31)

Given the strength of evidence of the association between poverty and the indicators of adverse childhood social conditions amongst the homeless, it is reasonable to conclude that poverty and homelessness are associated. Our conclusion is consistent with the proposition by van Leeuwen [15] that both the rich and the poor have the freedom to sleep on the streets at night, but the rich fail to take advantage of this freedom. By this proposition, van Leeuwen implies that the privileged do not have to live and sleep on the streets [15]. Van Leeuwen postulated that homelessness is a tragic condition that is the result of different causes, both structural (e.g., political-economical) and individual (e.g., addiction, mental illness, unemployment, and traumatic life histories) [15].

Our model illustrates how adverse childhood conditions impact on adults’ ability to gain the necessary tools and freedom to successfully participate in society [7]. Our model is consistent with findings by Mabhala et al. [58], which indicate that exposure to adverse childhood conditions erodes a person’s resilience to life challenges. Life stressors without positive coping mechanisms lead to the adoption of maladaptive behaviours, such as those listed in our model, causing people’s loss of employment and breakdown of relationships with those around them. Reducing poverty might be one strategy to reduce both ACEs and homelessness.

## 5. Discussion

This study simultaneously examined clusters of indicators of the social status of the parents of homeless people; the social conditions in which homeless people were raised; the ACEs amongst homeless people; and the immediate causes of homelessness. While several studies have examined these conditions, we have not found any that examined all at the same time. Furthermore, to the best of our knowledge, this is the first study that used two-step cluster analysis to classify social determinants of homelessness according to the order of predictor importance.

The clusters significantly associated with and most predictive of homelessness included the following indicator variables: criminal record, educational attainment, care history, single mother, living in care, sexual abuse, inappropriate sexual behaviour, emotional neglect and physical neglect, and drug or alcohol dependence.

The mean age of homeless people in this study was 39 years, with more males (63%) than females (37%); they were mostly unmarried (91%), and living in the street (31%), TA (21%) or hostel (19%). Our study’s demographic data is consistent with the UK government’s 2019 official statistics [59]. It is also consistent with similar studies from outside the UK, e.g., the longitudinal study of Magee et al. in three Canadian cities: two-thirds of the sample were male, one-third female, and an average age of 42.2 years [60].

In this study, the frequencies of self-reported poor health and health-damaging lifestyle were higher than expected—86% of participants smoked tobacco, 80% used drugs, 61.2% described their health as poor, and 69% visited a medical doctor in the last 6 weeks. These higher frequencies have been reported in many other homelessness studies [61,62]. For example, Sharman et al. sought to ascertain drug and alcohol misuse rates among homeless people in Westminster, London (UK). They found that 31.9% of participants used drugs, and 23.6% had alcohol dependence [62]. Similarly, in Los Angeles (USA), the Barman-Adhikari et al. study reported significantly high use of heroin (45.75% vs. 5.51%, *p* < 0.0001), marijuana (64.78% vs. 54.19%, *p* < 0.01), or injection drug (43.72% vs. 4.63%, *p* < 0.0001) [63]. In Khezri’s study in Iran, the most common substances used among homeless people were heroin (34.0%), alcohol (31.2%—despite alcohol being illegal in Iran), and methamphetamine (24.0%) [64].

This study revealed that homeless people’s parents had significantly different social status indicators from expectations. The particularly significant indicators were their educational attainment, marital status, employment status, criminal record, care history, and history of child neglect/abuse. Classification by predictor importance showed that the cluster of parents with a criminal record, care history, and child neglect/abuse was significantly associated with homelessness of their offspring. While several studies had reported the association between these indicators and homeless people themselves [65,66,67,68], no study was found that documented the frequencies and clustering of these indicators amongst the parents of homeless people.

This study shows that the following ACEs: living in care, being raised by a single mother, and leaving school before or at 16 were significantly higher than expected amongst participants. According to their predictor importance, clusters of ACEs associated with homelessness were sexual abuse, inappropriate sexual behaviour, emotional neglect, physical abuse by step-parent, and physical neglect. These findings are comparable with several studies that observed a high prevalence of ACEs among homeless people. For example, Spinelli et al. [7] examined the prevalence of and the factors associated with ACEs in a population-based sample (N = 350) of homeless individuals in Oakland, CA, USA. They found that the frequencies of suspension or expulsion from school, physical abuse as a child, sexual abuse as a child, physical violence as an adult, and sexual abuse as an adult were higher than expected [7]. In Canada, Barker et al. [32] also found that physical, emotional, and sexual abuse and physical and emotional neglect experiences were prevalent among at-risk street-involved youth. While many other studies examined and reported these homelessness indicators, no study was found that classified them according to predictor importance. We believe that such classification is useful for planning and prioritisation of interventions to prevent homelessness.

The two-step cluster analysis, and χ2 and post hoc tests, revealed that the most significant factors that led to becoming homeless were drug or alcohol dependence, eviction due to criminal activities, loss of job, and being imprisoned. The connection between loss of employment and engagement in maladaptive behaviour such as substance misuse, crime, and homelessness has been extensively reported [3,64]. Mabhala et al. argued that while reporting these observations could be seen as trite, what is pertinent is understanding the conditions within which these behaviours occurred [3,59,69]. Fry et al. proposed that poverty and homelessness are intertwined in that there is a high prevalence of poverty among the homeless [3]. Poverty sets up a chain of interactions amongst a range of social conditions—such as those identified above—that increase the likelihood of unfavourable outcomes, with homelessness at the extreme end of the chain.

## 6. Conclusions

We considered our results in conjunction with previous studies to conclude that poverty and homelessness are intertwined because there is a high prevalence of poverty among homeless people: interventions that support families to rise out of poverty may therefore also reduce entry into homelessness. Poverty sets up a chain of interaction amongst social conditions that increase the likelihood of unfavourable outcomes; homelessness is at the extreme end of the interaction chain.

## Figures and Tables

**Table 1:** Study objectives and corresponding variables.

Objective	Social Exposure Variables
1. To describe the health and demographic characteristics of homeless people.	- Age - Gender - Marital status - Military background - Nationality - Place of dwelling - Self-reported alcohol and smoking use - Self-reported drug use - Self-reported health status - Self-care - Use of health services - Types of illnesses - Use of prescription medication
2. To describe the social characteristics of the parents of homeless people.	- Education - Marital status - Employment - Criminal history - Looked after status - Child neglect/abuse
3. To describe the history of occurrence, frequency, and clustering of adverse social conditions among homeless people.	- Care history - Childhood living arrangement - Education attainments - Criminal history
4. To describe the occurrence, frequency, and clustering of adverse childhood experiences amongst homeless people.	- Verbal abuse - Physical abuse - Sexual abuse - Emotional abuse - Neglect - Family breakdown - Domestic violence - Exposure to drugs and alcohol - Exposure to crime
5. To describe the clustering of factors leading to homelessness.	- Relationship breakdown - Loss of income - Health - Crime

**Table 2:** Sums of the frequencies of participants’ characteristics, according to location of data collection.

Latent Variables	Drug Type	Indicator Variable	Chester	Liverpool	Manchester	Crewe
Variables			N = 57	N = 57	N = 35	N = 3
Gender		Female	10	23	8	2
		Male	47	34	27	1
Age		Mean	39.98	38.86	36.80	35.67
		Minimum	21	18	21	21
		Maximum	65	65	59	46
Marital status		Single	44	51	34	3
		Divorced	5	0	1	0
		Separated	2	0	0	0
		Married	2	3	0	0
		Civil partnership	4	2	0	0
		Widowed	0	1	0	0
Living arrangement		Hostel	11	22	6	0
		Street	11	26	21	0
		Covid 19 accommodation	0	8	5	1
		Temporary accommodation	11	0	0	2
		Other	14	1	3	0
Tobacco		Daily	46	40	32	3
		Occasionally	10	16	3	0
		Never	10	16	3	0
Drugs		Daily	45	43	32	3
		Occasionally	6	8	5	0
		Not anymore	1	0	0	0
		Never	5	3	7	0
Type of drug	NPS	Yes	3	2	0	0
		No	53	30	34	3
		Prefer not to say	1	3	1	1
	Heroin	Yes	22	17	13	1
		No	34	37	21	2
		Prefer not to say	1	3	1	0
	Spice	Yes	14	6	9	0
		No	42	48	21	2
		Prefer not to say	2	3	1	0
	Cocaine	Yes	21	31	13	0
		No	35	23	21	3
		Prefer not to say	1	3	1	0
	Marijuana	Yes	33	36	18	1
		No	23	18	15	2
		Prefer not to say	1	4	1	0

All cells containing figures are column sum values.

**Table 3:** The two-step cluster model that classifies the social indicators of the parents of homeless people according to the ratio of size, the average silhouette of the model, PImp values, χ2 values, and variable-specific Cramer’s V values.

Variables	Cluster Sizes	PImp	χ2 Value	Adj_*p* Value	Cramer’s V
Cluster 1	=(106) 67.1%				
Cluster 2	=(20) 13.2%				
Cluster 3	=(30) 19.7%				
Ratio of size	=5.10				
Average silhouette	=0.8				
Parents with a criminal record	–	1.00	231.105	0.001	0.9
Parents with care history	–	0.89	169.434	0.001	2
Parents with child neglect/abuse history	–	0.72	187.316	0.001	1.8

**Table 4:** Observed frequencies of indicators of homeless people’s social status compared with those expected if the null hypothesis is true.

Variables	Indicators	*fo*	*fe*	Z Values	χ2	*p*-Value	*Adj. p* Value
Childhood living arrangement (N = 152)					398.263158	0.001	0.001
Educational arrangement (N = 152)					432.868	0.001	0.001
Highest qualification (N = 152)					319.669	0.001	0.001
Childhood living arrangement	Biological parents	75	12.7	62.30	3881.29	0.001	0.001
	Sibling/s	4	12.7	−8.70	75.69	0.001	0.001
	Mother	19	12.7	6.30	39.69	0.001	0.001
	Other families	6	12.7	−6.70	44.89	0.001	0.001
	In care	29	12.7	16.30	265.69	0.001	0.001
	Father	1	12.7	−11.70	136.89	0.001	0.001
	By self	2	12.7	−10.70	114.49	0.001	0.001
	Step-parent	8	12.7	−4.700	22.09	0.023	0.260
	Street alone	2	12.7	−10.70	114.49	0.001	0.001
	Foster carer/s	4	12.7	−8.70	75.69	0.001	0.001
	Adoptive parent/s	1	12.7	−11.70	136.89	0.001	0.001
	Other arrangements	1	12.7	−11.70	136.89	0.001	0.001
Educational arrangement	Mainstream school	110	21.7	88.30	7796.89	0.001	0.001
	Other educational arrangements	2	21.7	−19.70	388.09	0.001	0.001
	Special school	13	21.7	−8.70	75.69	0.001	0.001
	Pupil referral unit	20	21.7	−1.70	2.89	0.822	4.94
	Other specialist units	5	21.7	−16.70	278.89	0.001	0.001
	Home-schooled	1	21.7	−20.70	428.49	0.001	0.001
	No formal education	1	21.7	−20.70	428.49	0.001	0.001
Highest qualification	Left school before 16	80	21.6	58.40	3410.56	0.001	0.001
	Left school at 16	61	21.6	39.40	1552.36	0.001	0.001
	College/further education	5	21.6	−16.60	275.56	0.001	0.001
	College diploma	2	21.6	−19.60	384.16	0.001	0.001
	University degree	1	21.6	−20.60	424.36	0.001	0.001
	Other	1	21.6	−20.60	424.36	0.001	0.001
	No formal education	1	21.6	−20.60	424.36	0.001	0.001
**Total**		**152**					

**Table 5:** The two-step cluster model that classifies the adverse childhood experiences amongst homeless people according to the ratio of size, the average silhouette of the model, PImp values, χ2 values, and variable-specific Cramer’s V values.

Variables	Cluster Sizes	PImp	χ2 Value	Adj_*p* Value	Cramer’s V
Cluster 1	=(32) 21.1%	–	349.690	0.001	1.0
Cluster 2	=(26) 16.4%	–	660.490	0.001	1.5
Cluster 3	=(95) 62.5%	–	1962.490	0.001	2.5
Ratio of size	=3.8	–	-	–	–
Average silhouette	=0.8	–	-	–	–
Sexual abuse	–	1.00	220.684	0.001	0.7
Inappropriate sexual behaviour	–	0.99	207.737	0.001	0.7
Emotional neglect	–	0.70	181.671	0.001	0.7
Physical abuse by step-parent	–	0.65	195.882	0.001	0.8
Physical neglect	–	0.64	205.632	0.001	0.8

**Table 6:** The two-step cluster model that classifies the factors leading to homelessness according to the ratio of size, an average silhouette of the model, PImp values, χ2 values, and variable-specific Cramer’s V values.

Variables	Cluster Sizes	PImp	χ2 Value	Adj_*p* Value	Cramer’s V
Cluster 1	=(46) 30.3%				
Cluster 2	=(42) 27.6%				
Cluster 3	=(64) 42.1%				
Ratio of size	=1.5				
Average silhouette	=0.8				
Drug and or alcohol dependence	–	1.00	0.026	0.871	0.04
Eviction due to your criminal activities	–	0.55	79.605	0.001	0.72
Loss of job	–	0.52	82.526	0.001	0.73
Went to prison		0.40	94.737	0.001	0.79

## Data Availability

The data presented in this study are available on request from the corresponding author. The data are not publicly available due to the sensitive nature of subject matter.
